# Thoracoscopic Repair of Congenital Diaphragmatic Hernia in Preterm Neonate at 1 Kilogram

**DOI:** 10.1055/s-0040-1721473

**Published:** 2021-01-28

**Authors:** Muhammad Choudhry, Simona Rusu, Peter Brooks, Enitan Ogundipe, Shu-Ling Chuang

**Affiliations:** 1 Department of Pediatric Surgery, Chelsea and Westminster Hospital National Health Service Foundation Trust, London, United Kingdom of Great Britain and Northern Ireland; 2 Department of Anaesthesia, Chelsea and Westminster Hospital National Health Service Foundation Trust, London, United Kingdom of Great Britain and Northern Ireland; 3 Department of Neonatology, Chelsea and Westminster Hospital National Health Service Foundation Trust, London, United Kingdom of Great Britain and Northern Ireland

**Keywords:** congenital diaphragmatic hernia, thoracoscopic repair, premature

## Abstract

We report the first successful primary thoracoscopic repair of congenital diaphragmatic hernia (CDH) in a preterm infant born at 28 weeks of gestation weighing 1,043 g. Left-sided CDH was incidentally diagnosed on postnatal chest X-ray on day 1. The neonate subsequently underwent thoracoscopic repair with primary closure of the defect on day 8 weighing 1,150 g. Intraoperative arterial blood gas monitoring including end tidal carbon-dioxide remained within normal range throughout. Postoperative recovery was uneventful. One year neurodevelopmental outcome was normal for age with no CDH recurrence.

## Introduction

The first pediatric thoracoscopic repair of congenital diaphragmatic hernia (CDH) was reported in 1995 in a 16-year-old boy 1 and 2001 in a 9-month-old infant. 2 Currently, this approach has been widely used in neonates, full-term and premature neonates with acceptable outcomes. Minimally invasive surgery (MIS) is slowly becoming an accepted procedure at a younger gestational age and lower weight neonates for different conditions; however, challenges still arise in low birth weight neonates. We report a case of successful thoracoscopic repair of CDH in an 8-day old, 1kg premature neonate born at 28 weeks of gestational age.

## Case Report

**Fig. 1:**
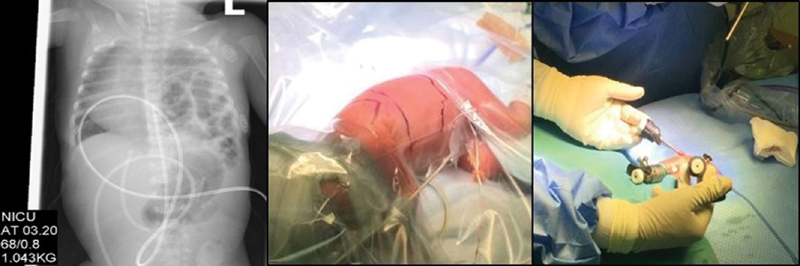
Postnatal X-ray, left-sided congenital diaphragmatic hernia. Patient position and placement of the ports. NICU, neonatal intensive care unit.

A female infant was transferred from ex-utero to our neonatal unit on day 1 of her life with postnatal diagnosis of left-sided CDH. Maternal antenatal care was overseas and antenatal ultrasound scans performed there were all reported as normal. The neonate was born at 28 ^6/7^ weeks of gestation via emergency caesarean section weighing 1,043 g. She was appropriately grown for gestational age. Apgar's scores at birth were 8, 9, and 9 at 1, 5, and 10 minutes, respectively. At 20 minutes, she was intubated, received surfactant therapy for persistent respiratory distress, and placed on a ventilator at the local neonatal unit prior to transfer. The postintubation chest radiograph revealed the left-sided CDH ( Fig. 1) consistent with the clinical examination. After transfer to our unit, her ventilatory support remained conventional initially and later changed to high-frequency oscillatory ventilation (HFOV). Inotropic support was required for 5 days and consisted of adrenaline, noradrenaline, and dopamine (all three required). The doses varied between 5 and 20 µg/kg/hour and were constantly adapted to maintain the blood pressure within the normal range. There was no pulmonary hypertension and nitric oxide was not required preoperatively. Further examination of the neonate confirmed a phenotypically normal infant with no dysmorphic features or other congenital abnormalities. An echocardiogram confirmed a structurally normal heart.

On day 8, when she was clinically stable to be operated for the left CDH repair (on conventional ventilation), a decision to undertake a thoracoscopic repair was agreed in a planned preoperative multidisciplinary discussion involving neonatologists, anesthetists, and the surgeons. Parents were informed in detail and their consent was obtained. She weighed 1,150 g at the time of surgical procedure.

The patient was placed in the right lateral decubitus position. One 5-mm camera port was inserted at the tip of the scapula for a 0-degree thoracoscope, and two further 3-mm instrument ports were inserted under direct thoracoscopic visualization at 4th intercostal space in the anterior and posterior axillary line ( Fig. 1 ). Carbon dioxide (CO _2_ ) insufflation pressure was initially kept at 4 mm Hg until the reduction of the herniated organs (small and large bowel, stomach, and spleen), and then reduced and later switched off. Findings after reduction were of a posterolateral diaphragmatic defect with good muscle edges, a small left lung, and no hernia sac. The diaphragmatic defect was closed primarily using three tension-free interrupted 3/0 Ethibond sutures between the anterior and posterior muscle edge with two additional posterolateral sutures encircling the rib. The port site wounds were closed using absorbable 4/0 Vicryl and skin glue. No chest drain was placed and pressure dressings were applied to prevent surgical emphysema. The entire duration of the procedure from the first skin cut to closure was 110 minutes. Any brief stops or interruptions during the procedure for any blood gas monitoring/testing or relating to instruments were inclusive within this time and was not deducted.

There was a close monitoring of CO _2_ levels, cerebral oxygenation, and arterial blood gas throughout the operative procedure. She maintained an average end tidal CO _2_ of 31.5 mm Hg, ranging between 27.75 and 38.25 mm Hg and average arterial oxygen saturation of 99%. Following insufflation, her arterial CO _2_ increased from 47.25 to 75 mm Hg. Her regional cerebral oxygen saturation dropped below 55%. Promptly, minute ventilation and inspired oxygen were increased and a fluid bolus was given. A decision was made to discontinue insufflation of CO _2_ which allowed the inflation pressure dropped to zero. The regional cerebral oxygen saturation steadily increased to above the safety threshold over 5 minutes; there was a short respiratory acidosis increased to a pH of 7.03 and an arterial CO _2_ of 89.25 mm Hg. This was reversed on reinflation of the collapsed lung. No inotropic support was required during the procedure.

She was on conventional ventilation for 6 days postoperatively and extubated on to continuous positive airway pressure (CPAP) for a further 8 days. Initially, she received total parenteral nutrition and enteral feeds commenced on day 15 after birth. She was transferred back to her local hospital on day 24 after birth for the establishment of oral feeds and weight monitoring as appropriate for a preterm neonate. She developed bronchiolitis requiring nasal cannula oxygen and intravenous antibiotics for a week from which she made a good recovery. She was discharged home on day 50 after birth at a corrected gestational age of 36 weeks with a weight of 2.1 kg.

**Fig. 2:**
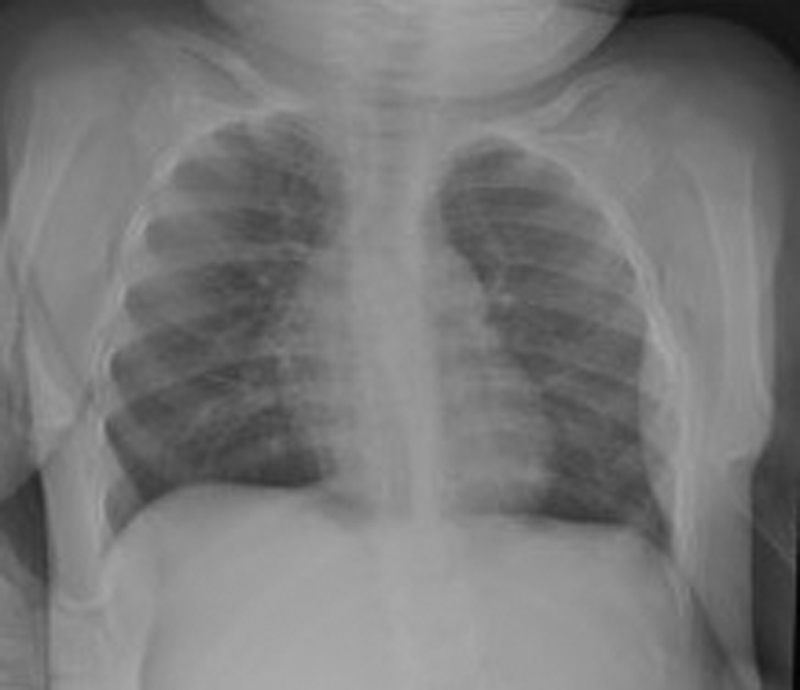
Chest X-ray at 1 year postoperative.

Her 1-year neurodevelopmental assessment was normal for age with good growth; weight was 9.15 kg (75th–91st centile) and head circumference was 46 cm (91st–98th centile). Chest X-ray showed no recurrence of CDH ( Fig. 2 ).

## Discussion

Thoracoscopic approach for CDH has some advantages over the traditional open technique, especially in decreasing pain and incisional related morbidity, shorter recovery, and hospitalization. 3 4 This technique provides magnified visualization and relatively easy reduction of herniated abdominal viscera. By contrast, particular concerns arise due to physiological stress associated with MIS and acidosis due to retained CO _2_ in infants. CO _2_ insufflation during the procedure may cause hypotension, tachycardia, and dynamic fluctuations in cardiac index, especially in neonates with associated cardiac abnormalities or significant pulmonary hypoplasia. 5 6 These possible complications could prompt conversion to open surgery. Gomes Ferreira et al evaluated the factors that would determine the failure of primary thoracoscopic repair and concluded that the severity of pulmonary hypertension and the capacity to tolerate a capnothorax that plays an important role. 7 Close monitoring during the procedure of the arterial blood gases is important in weighing the risks and benefits of MIS. Consequently, a comprehensive discussion with the neonatologist and anesthetist prior to embarking on the procedure is essential.

At our unit, all neonates going for major or complex surgical procedures are routinely discussed with the teams involved (neonatologist, surgeon, and anesthetist) preoperatively as a routine. At these meetings, anticipated issues, approach, and intending procedures including risks are discussed. It is not common for the neonatologist to be present in the operation theater at the time of surgery; however, they are available to be called in if required. The same preoperative meeting was held, and details discussed in the case of the above reported child. The main anesthetist with his associates (other anesthetists) were managing anesthesia and monitoring have asked for short periods of stops and rests during this case. The anesthetic team was happy to continue with these short breaks/rests and did not suggest converting this procedure to open due to brief periods of acidosis and dropping saturations encountered during the surgery. As mentioned in the case report, the initial gas pressure was 4 mm Hg and was reduced to minimal and later switched off once contents were fully reduced prior to the defect repair. As a routine, we tend to recommend conversion of the procedure to open if gases stay continuously abnormal or otherwise advised by the anesthetist.

In addition to the possible physiological challenges detailed above, performing the repair in a limited small space in itself poses a technical challenge, despite utilizing appropriately sized instruments. In this case, a 0-degree scope was used (lead surgeon preference) at the start of the procedure and continued with the same as it provided an end on vision. However, a 30-degree scope was available and ready to be used if visualization was an issue or thoracoscopic patch repair would have been required to visualize rims and corners. Both scopes are always part of our set and changed accordingly if any issues with the visual field in either case. Recent case series reports have shown thoracoscopic technique is safe and feasible. Wall et al reported their MIS experience in patients weighing less than 3 kg (1.5–3 kg), and three of their four cases of CDH required conversion to an open procedure due to the need of patch repair 8 ; they recommended MIS in infants weighing >1.8 kg due to potential increased operative risk associated with extra operative time needed for patch repair using MIS.

In this case report, antenatal scans were performed in Pakistan and no pictures were available to review and the diagnosis that the CDH was made postnatally. This largely depends on the expertise of the obstetrician, as this condition is not antenatally diagnosed in all cases and accuracy varies among different centers. Embryological defects do not form toward the end of the pregnancy; however, a small defect can present with minimal contents or contents which go in and out of the chest at times. Presence of lung hypoplasia in our case suggests that contents may have been there for longer. Lung was found to be hypoplastic in this case, as one would expect antenatally diagnosed CDH and that was not coming in the operating field during the repair in spite of no gas insufflation. In addition, it did not occupy the whole empty space at the end of the defect repair when overinflated to check for any parenchymal leaks prior to wound closures.

Keijzer et al reported 23 cases of thoracoscopic repair in infants with a birth weight between 1.6 and 4.1 kg; eight required patch repair and six were converted to open surgery due to large size of the defect or difficulty in reducing the herniated organs. 9 There are several articles that discussed the criteria for selecting CDH cases suitable for MIS in neonates and these largely consist of cardiovascular stability with no persistent pulmonary hypertension, no need for nitric oxide during surgery and no need for extracorporeal membrane oxygenation (ECMO). 3 10

## Conclusion

Primary thoracoscopic repair of congenital diaphragmatic hernia in a low birth weight premature neonate is achievable with a successful surgical outcome. However, the decision to proceed with MIS in low birth weight and gestation neonates should be discussed and planned with anesthetist and neonatologist prior to the procedure.

## Notes

New Insights and the Importance for the Pediatric Surgeon Minimally invasive surgery is becoming an accepted procedure at younger gestational age and lower weight groups for different surgical conditions. The thoracoscopic approach of the congenital diaphragmatic hernia is achievable with successful outcome in premature newborn also.
